# DNA testing for high risk of prostate cancer

**DOI:** 10.1186/1897-4287-10-S1-A12

**Published:** 2012-01-12

**Authors:** Dominika Wokołorczyk

**Affiliations:** 1 International Hereditary Cancer Center, Pomeranian Medical University, Szczecin, Poland

## 

Association studies of candidate genes in DNA repair and cell cycle control pathways identified mutations associated with a susceptibility to prostate cancer in BRCA1, BRCA2, CHEK2, NBS1 and BRIP1 genes. Mutations in these genes confer 1.5- to 6-fold increase in the risk of prostate cancer. In general, the risks associated with these mutations are higher in carriers who report family history of prostate cancer (the risk increased 3- to 15-fold).

Our studies confirm that rare mutations in DNA damage repair genes are associated with a predisposition to prostate cancer. Specific mutations in NBS1, BRCA1 and CHEK2 genes are associated with 1.6- to 4.6-fold increased risk for prostate cancer in the Polish population. The risk is higher, increased approximately 5 - 15 fold, in carriers who report prostate cancer in at least one first and/or second degree relative.

In the past three years, new DNA markers of low penetration for prostate cancer were identified by GWAS studies. Of these markers, the strongest association with disease risk was seen for markers of chromosome 8q24. We analyzed how markers of this region influence prostate cancer risk in a in a series of cases and controls from Poland. Single markers of 8q24 were associated with a low penetrance for prostate cancer - approximately 1.5- fold increased risk (ORs ranged from 1.4 to 1.6). Carriers of two different markers had the risk increased on average by 2,5-fold. Carriers of risk alleles of three markers had on average 6-fold increased risk. Carriers of five markers of 8q24 had an odd ratio of 10.7 for prostate cancer (95% CI 3.3 - 36).

